# Issues with the Swedish e-prescribing system – An analysis of health information technology-related incident reports using an existing classification system

**DOI:** 10.1177/20552076221131139

**Published:** 2022-10-11

**Authors:** Md Shafiqur Rahman Jabin, Tora Hammar

**Affiliations:** Department of Medicine and Optometry, Faculty of Health and Life Sciences, eHealth Institute, 4180 Linnaeus University, Kalmar, Sweden

**Keywords:** System design, system functionality, system interface, software problem, electronic prescription, medication management, human factor, technical factor, healthcare quality, patient safety

## Abstract

**Objective:**

To identify issues with the Swedish e-prescribing system and devise a set of recommendations to overcome the identified challenges.

**Methods:**

A number of health information technology-related incidents were collected retrospectively from various sources using purposive and snowball sampling. A search term containing five keywords was used to identify the electronic prescription-related incidents. The identified incidents (*n* = 24) were subjected to an existing framework, i.e., the Health Information Technology Classification System. Special attention was paid to the software-related issues, which were analysed using thematic analysis.

**Results:**

Several types of software-related issues (*n* = 22) were identified: system configuration, interface with other software systems or components, software functionality, data storage and backup, record migration, software not accessible, and network/server down or slow. Both human and technical factors contributed to these incidents, including prescriptions not cancelled actively, drug handling errors, software programming errors, and system updates/upgrades. These software problems led to various consequences, such as incidents affecting multiple patients’ care management, delays in patient care, and risks of serious deterioration of health. Several temporary initiatives or administrative adjustments, for instance, cover letters to patients and local strategies, were used to overcome some of these challenges.

**Conclusions:**

This study provides insights into the challenges related to the e-prescribing system, contributing factors, consequences, and actions taken to mitigate those risks. Therefore, healthcare organisations using the e-prescribing system should adopt the provided recommendations to minimise the risks of design and developmental challenges, implementation and use-related issues, and the problems related to monitoring, evaluation, and optimisation.

## Background

Health information technology (HIT) holds the potential to complement the safe and high-quality delivery of care by reducing adverse events^1^ and improving accuracy.^2^ HIT has improved several dimensions of healthcare quality, such as increasing efficiency,^3^ effectiveness,^4^ and empowering patients.^5^ However, it is evident from several studies that successful deployment of HIT systems in one setting may fail miserably in others, a problem well documented in implementation science.^6,7^ In Sweden, electronic prescribing (e-prescribing) has been considered a complex, sociotechnical system integrated with other systems, such as Electronic Medical Records (EMR) and dispensing systems at pharmacies. For the convenience of the reader, the term e-prescribing system is used in this study to encompass the sociotechnical system related to electronic prescriptions and the people involved in these systems.

An e-prescribing system has been proposed as an essential tool to improve quality.^8–10^ However, weaknesses with paper prescriptions are not necessarily solved, and e-prescribing may even create new errors.^11–13^ Sweden introduced the world's first electronic prescription for outpatients and is still one of the leading countries, with more than 99% of all prescriptions being electronic.^14,15^ Since 2005, patients have been able to store their valid prescriptions electronically in the Swedish national prescription repository.^9,16^ In healthcare, EMR is used for documentation as well as e-prescribing. However, many EMR systems are used in Swedish healthcare with varying electronic prescription functions and designs.^17^ The different regions in Sweden do not share the same EMR, and information is not automatically transferred between the different EMRs. Multi-dose drug dispensing is a service in which patients receive their medication machine-packed into a unit dose sachet for each time of administration.^18^ E-prescribing for multi-dose drug dispensing patients is primarily handled using a separate web-based system, and information is usually not automatically transferred to the medication list in the EMR.

Even though Swedish physicians have generally shown satisfaction with the overall integration of the EMR and e-prescribing system, there remained identified system deficiencies.^17,19,20^ Several studies have reported discrepancies between EMR, the Swedish national prescription repository, and the current medications reported by the patients.^21,22^ Due to a lack of electronic coordination and interoperability, these errors may occur at any phase of the electronic prescription process, such as transmission, storage, and dispensing.^17^ Several studies describe discrepancies between the medication lists and information used in health care, at community pharmacies and by patients in Sweden.^20–22^ Another recent study indicated that around 23% of large-scale events in Swedish healthcare were associated with e-prescribing systems.^23^ These e-prescribing problems can have serious consequences, which can range from inconvenience and workflow interruptions to patient harm.^21^

### An overview of the e-prescribing system in Sweden

**Figure 1.**
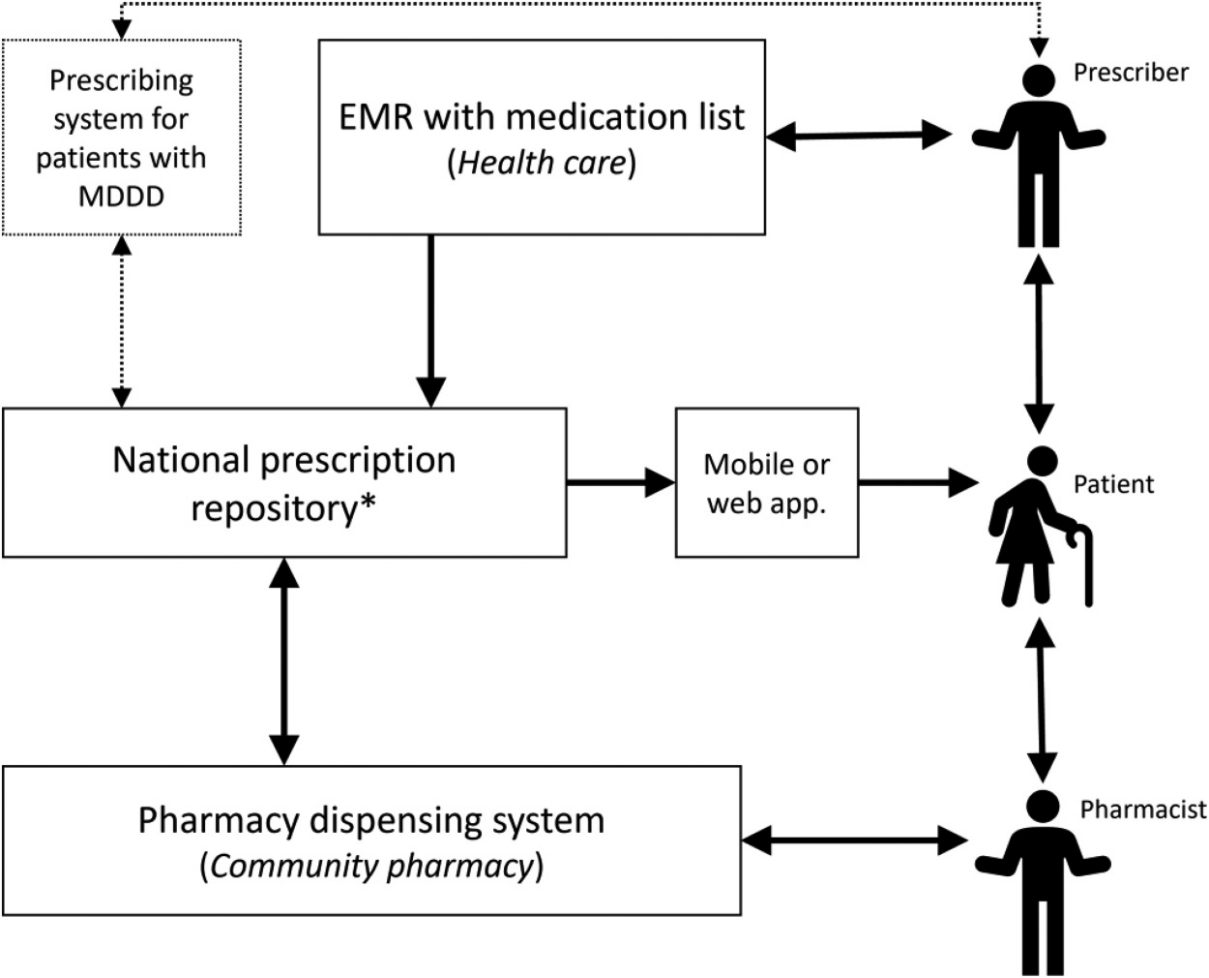
An overview of the e-prescribing system in Sweden. * The national prescription repository was replaced with the National Medication List in May 2021 (after data collection in this study). EMR: electronic medical record; MDDD: multi-dose drug dispensing.

Sweden is currently working on implementing a new National Medication List (In Swedish: nationella läkemedelslistan, NLL) to improve many of the issues in the medication management process, such as errors and discrepancies between different medication lists. A new law (*Lag (2018:1212) om nationell läkemedelslista*) came into force on 1 May 2021.^15^ However, data on incidents in this paper were collected before the new law was in place. A schematic representation (see Figure 1) and a description of e-prescribing in Sweden have been provided to better understand the integrated function of the prescribing system and the EMR.

The prescribers can prescribe medication via a prescribing module in the EMR system. The prescription is then transferred to the national prescription repository, where it can be stored through the entire period of its validity to collect medication several times. Prescribers can cancel their prescriptions if their EMR system supports this function, but they have not been allowed to view the patient's prescriptions stored in the prescription repository due to legal reasons. Prescribing for patients with multi-dose drug dispensing is managed in a separate system, usually not linked to the EMR. The medication can be dispensed at any Swedish pharmacy from the prescription repository. Upon request from patients, pharmacists can view and dispense patients’ prescriptions via their dispensing system. Patients can also view their electronic prescriptions via mobile applications or web pages using digital authentication. The national prescription repository was replaced with the National Medication List in May 2021, and prescribers are now allowed to view this information.

### Importance of incident reporting and a classification system

Challenges and issues related to electronic prescribing have been reported in many countries, where the situation in other Nordic countries is similar to Sweden.^24–26^ Many studies have been conducted using various methods, for instance, cross-sectional studies.^24,27,28^ These studies showed various aspects of electronic prescriptions, such as the impact of polypharmacy and drug-drug interactions among older people,^27^ the application of Swedish national indicators improving drug prescribing,^29^ and how potentially inappropriate medication affected older patients.^24^ However, the Nordic countries have not reported studies on the imbalance between safety and quality by using incident reports recorded in daily clinical practice.

In collaboration with the Swedish eHealth Agency and the Swedish Authority for Privacy Protection, the Swedish Medical Product Agency (MPA) has taken the initiative to implement an incident alert on the National Medical Information (NMI) systems. This capacity-enhancing action has contributed to promising and improved healthcare by highlighting the disproportion between healthcare quality, patient safety, and throughput. Healthcare professionals are encouraged to report incidents to their respective county councils’ existing digital reporting systems.^30^ Despite the effort made by the MPA and NMI to reduce the risks of incidents that occur in day-to-day Swedish healthcare due to medical devices and the HIT systems, it is still not customary to research these incident reports.

Using a classification system in data analysis is effective and efficient. An existing classification system is usually generated based on a large amount of data from various types of research and can be placed for progressive tests before its broad application.^31^ A classification system tailored to HIT (HIT-CS) was developed by Magrabi et al.^32^ to identify, characterise, and address the issues arising from HIT in healthcare. This classification system adopted a ‘bottom-up’ approach for categorising HIT-related issues. This classification system can categorise problems based on human or technical problems, whereas technical issues can be divided into hardware and software-related issues. A single incident can be categorised into more than one incident type or characteristic. The HIT-CS also offers to identify the contributing factors and the consequences of the incidents that occurred.^32^

The information can be captured about low-frequency incidents in day-to-day clinical practice, with the potential for specific issues being able to be characterised with the help of an existing framework, i.e., HIT-CS, which may not be affordable with a conventional prospective study design. Studies using incident reports, in particular, have the potential for specific problems to be identified and characterised, such as problems arising from unintended interactions between human and HIT systems in practice.^33^ Anecdotally, the healthcare organisations in Sweden are confronting similar incidents as those reported in the USA,^34^ the UK,^32^ and Australia.^35,36^ Therefore, we intended to collect, classify and analyse incident reports related to e-prescribing systems that would help devise preventive and corrective strategies as recommendations.

### Study aim

Multiple research studies have been conducted on the e-prescribing system in Sweden. Such studies included electronic prescription problems before dispensing,^37^ experience in the use of electronic prescriptions,^38^ implementing regionally shared medication lists,^20^ experience with a new dispensing system,^39^ discrepancies between the EMR and e-prescribing system,^21,40,41^ and physicians’ attitudes towards e-prescribing.^17^ However, no research has been conducted on the study of incident reports and various HIT-related problems of the e-prescribing system. Hence, there is a need for qualitative analysis to explore the issues involved with the e-prescribing system using the HIT-CS by studying the incident reports.

This research study examines the sources of information about what went wrong and how they went wrong in the day-to-day clinical practice of Swedish healthcare associated with electronic prescriptions. The study concentrates on the HIT incidents depicting the challenges encountered by the e-prescribing systems through the lens of HIT-CS and thematic analysis. The study explores the following research questions:

1. What are the reported issues involving the e-prescribing system in Sweden?
2. How did human and technical factors contribute to the issues related to the e-prescribing system?
3. What were the consequences of those issues related to the e-prescribing system, and what actions were taken to mitigate patient safety risks?
4. What would potential solutions be to reduce the risks associated with the e-prescribing system?

## Methods

### Data collection

Sources of information about things that had gone wrong associated with HIT in Swedish healthcare were identified. The incidents were retrospectively collected from various sources, such as interviews through written responses, telephone conferences, and three small sets of already existing digital incident databases. The retrospectively collected incident reports were considered to identify incidents related to electronic prescriptions.

The total sample of incidents was collected using multiple approaches, such as purposive and snowball sampling, targeting a range of healthcare professionals. The target population included physicians, nurses, medical engineers, and healthcare quality managers from 21 regions. A set of four interview questionnaires were used to collect incidents (see Box 1) for written responses and telephone conversations. The participants had the choice of replying to interview questions either via email or through semi-structured telephone interviews. The participants could otherwise provide a set of retrospectively collected incident reports from their local incident database. No structured format was used to collect incident reports from the existing databases, except for a formal request to the target groups.

A total of 55 participants were contacted using the purposive sample, of which only five responded. Later, 19 additional participants were reached due to limited response using snowball sampling, of which ten participated in the study. Therefore, a total of 74 participants were contacted, of which 15 responded. The 15 participants comprised six physicians, four medical engineers, three quality managers, and two nurses from a total of five regions in Sweden, i.e., the regions of Kalmar, Kronoberg, Stockholm, Uppsala, and Gävleborg. Three participants provided three sets of retrospectively collected incident reports (26 + 36 + 14 = 78) from their local database; the rest 12 respondents supplied 15 incidents through written responses and five through telephone interviews. Of 15 respondents, 98 incident reports were collected both in English and Swedish. The Swedish reports were translated into English by a linguistic expert, having proficiencies in both languages.

Of 98 incidents, 95 incidents were included, and three were excluded due to inadequate information. Of 95 included incidents, 77 comprised retrospectively collected incident reports from three voluntary incident reporting systems, and the rest (*n* = 18) were collected through interviews (written and telephone). Of 18 incidents, 13 comprised written responses, and the rest (*n* = 5) were through telephone interviews. The total sample of the incident reports ranged 5-year time period, from January 2016 to May 2021.

Box 1. Interview questionnaire

Questionnaire

1. Would you please briefly describe an eHealth-related incident/adverse event you have experienced?
2. What were the contributing factors leading to that incident/adverse event?
3. How did that incident/adverse event affect the patients, healthcare professionals, or the organisation?
4. What actions were taken by the healthcare team or the organisation to prevent or minimise that incident/adverse event?

### Data analysis

The incident reports collected through various means were in the form of free-text narratives, which were then aggregated for the purpose of analysis. A search term containing five keywords – drug, prescription, medicine, pharmaceutical, and medication, was used to identify the electronic prescription-related HIT incidents. The keywords were selected based on the iterative reading of the total sample of incidents to identify the relevant features of the narrative texts. Once the targeted incidents were identified, the rest of the incidents were re-read to confirm the validity of the search terms. This study design, i.e., a keyword search, is advantageous when there is a large volume of qualitative data, which are free-text narratives. A set of keywords can be considered indicative of incidents involving the research question, in this case, e-prescribing systems, which may be subjected to an appropriate classification system, in this respect, the HIT-CS.^36,42^

Using the keywords, 24 electronic prescription-related incidents were identified (see Table 1), which were then subjected to an existing framework, i.e., the HIT-CS. Since an incident can be categorised into more than one type of issue, a total of 47 issues were identified. Of these 47 issues, 22 were software-related, 20 had machine-related problems, and five had human or use-related issues (see Table 2). Special attention was paid to the software-related issues; however, no hardware-related problems were identified.

**Table 1.** Characteristics of identified incidents related to electronic prescription.

Incident report (IR) no.	Sources of information collected from	Drug	Incident reports that contained the keywords			
			Prescription	Medicine	Pharmaceutical	Medication
IR_1_	Existing database					✓
IR_2_	Written response	✓	✓	✓	✓	
IR_3_	Written response	✓				
IR_4_	Written response	✓	✓	✓		✓
IR_5_	Written response	✓	✓		✓	
IR_6_	Telephone interview			✓		
IR_7_	Telephone interview	✓	✓	✓		
IR_8_	Existing database	✓				
IR_9_	Existing database	✓	✓			
IR_10_	Existing database	✓		✓		
IR_11_	Existing database		✓			
IR_12_	Existing database				✓	
IR_13_	Existing database	✓	✓	✓		
IR_14_	Existing database	✓			✓	
IR_15_	Existing database		✓			
IR_16_	Existing database		✓			✓
IR_17_	Existing database	✓				
IR_18_	Existing database	✓				
IR_19_	Existing database		✓			
IR_20_	Existing database	✓	✓			
IR_21_	Existing database	✓				
IR_22_	Existing database	✓	✓	✓		
IR_23_	Existing database				✓	
IR_24_	Existing database			✓		

**Table 2.** Coding of the identified incidents related to electronic prescriptions and their short description of the core issues.

Incident report no.	Human/use-related issue	Machine (technical) related issue	Software (technical) related issue	Short description of the core issues
IR_1_	Wrong entry or retrieval			Healthcare staff incorrectly deleted patient information about blood pressure medication resulting in the wrong medication for patients
IR_2_		Wrong output	Interface with other software systems or components	The lack of a link between the medication list in EMR and the National prescription repository caused significant risks to patient safety
IR_3_		Delayed output	Interface with other software systems or components	Lack of coordination between the clinical care support (administrative) system and the e-prescribing system led to the manual work of the healthcare staff
IR_4_		Delayed output	System configuration	The system is not designed to cancel the old prescriptions resulting in patients inadvertently continuing with non-current drugs
IR_5_	Did not enter or retrieve	Wrong output	System configuration	The list of old medicines was not set up in a way that it became automatically cancelled when new medicines were prescribed for the patient, which created confusion for the staff
IR_6_		No output	Software functionality	Faulty software did not transfer sickness certificates and messages related to patient medication affecting patient documentation
IR_7_		Wrong output	Software functionality	Electronic prescriptions went wrong for renewed prescriptions due to functional errors resulting in confusion and frustrations among staff
IR_8_		No output	Software functionality	Information about patient medication from the medical record disappeared because of software malfunction, causing delays in patient treatment
IR_9_		No output	Software functionality	A number of prescriptions for inpatient care have disappeared for three patients (system dysfunction), affecting multiple patients’ care management
IR_10_	Wrong entry or retrieval			An incorrect end date was given for the automatic drug dispensing, resulting in delays in the care delivery
IR_11_		Delayed output	Software functionality	A software function in X (a system for patient care documents, including prescriptions) caused delays in message transfer and therefore delay in patient care
IR_12_		Delayed output	System configuration	The ‘benefit terms’ were not set up to be displayed in the new version of the application of the pharmaceutical module resulting in an additional workload for the healthcare professionals
IR_13_		Wrong output	System configuration	Prescription and administration (within the hospital) views were separate. Physicians and pharmacists did not have the same view leading to staff confusion
IR_14_		Partial output	Interface with other software systems or components	Patient change in the Care Portal did not coordinate with the current patient displayed in the pharmaceutical module, causing delays in patient treatment
IR_15_		No Output	Data storage & back-up	Prescriptions were missing from the mini-backup affecting the entire organisation, including health IT staff, and the quality of healthcare
IR_16_	Did not enter or retrieve	No output	Interface with other software systems or components	Thirty medical prescriptions were not sent from one system to another. 13 of these marked the warning message and disapproved of the prescription. The remaining 17 did not interpret the message correctly, causing risks to patient safety
IR_17_			System configuration	The root cause of the error was that the system was not configured according to instructions, creating the risk of incorrect dosing of the drugs
IR_18_		Partial output	Interface with other software systems or components	Patient change in the Care Portal did not correspond to the current patient displayed in the Care Documentation where the patient's medication information existed, causing staff confusion
IR_19_		Partial output	Software not accessible	A software conversion programme was not accessible to handle a vaccination file completely without valid vaccination registration attributing to delays in patient care
IR_20_		Partial output	Interface with other software systems or components	When a prescriber changes a prescription with a ‘prescription type without time’ to ‘prescription type with time’ via Dosage Overview, the administered dose is no longer visible in the administered list but in the administered dialogue exposing the patients to the risks of serious deterioration of health
IR_21_		No output	Network/server down or slow	A network down of the electronic prescribing system led to the medicine list not working for a longer period
IR_22_		No output	Software functionality	The ‘exit’ function of the medicine list was not functioning after making the latest change in the system, which affected healthcare quality
IR_23_	Did not enter or retrieve	No output		The sample responses from the last sampling were not sent, and the user did not receive information in the pharmaceutical module, placing the patients at risk to care delivery
IR_24_			System configuration	The system was not configured to give a warning for registered hypersensitivity information about medicine causing risks of serious deterioration of patients’ health

IR: incident report; EMR: electronic medical records; IT: information technology.

The incidents entailing software challenges (*n* = 22) were then analysed using thematic analysis – the themes were generated through the existing framework of HIT-CS. The coding and theme extraction analyses were regulated and managed on a semantic level. The explicit content of the interview was considered without making any assumptions about the latent underpinnings of the data. Table 2 briefly describes each incident's core issues and the HIT-CS coding for ‘types of issues. The contributing (human versus technical) factors, consequences, and actions taken to mitigate the risks were subjected to theme extraction analyses, which were regulated and managed on a semantic level. The HIT-CS was not applied to them because not all incidents could provide adequate information to be classified.

The principal investigator performed both the deductive (HIT-CS) and inductive (thematic) analyses for the 24 included incidents, and the second coder cross-checked each incident coding. The coders came to a harmonious agreement between themselves if there was any disagreement.

## Results

The results are presented under the rubric of types of software-related issues following the HIT-CS and illuminated in additional detail, such as the effects of the incidents extracted from the empirical data. In addition, the contributing (human versus technical) factors, consequences, and actions taken to mitigate the risks are presented to understand the underlying mechanisms and impact of such outcomes associated with these incidents.

### System configuration

A common concern was noticed when a change in prescription (with a new one) occurred; the old list remained in the e-prescribing system if someone (e.g., a pharmacist) did not actively cancel the previous (outdated) prescription. The e-prescribing system was not set up to cancel any non-current medication on the list in case of any prescription change. As a result, the patient might inadvertently continue with previous, non-current drugs if the prior prescription had not been actively cancelled. The patients did not always remember what had been decided and were often confused because different drug lists in two other systems existed and provided different messages, such as a list of available prescriptions versus the current prescription list from the last contact with the physician. For example, an old patient who came to the doctor because he felt dizzy and turned out to be over-treated with blood pressure medication received a lower dose and continued with both the high and low (new) doses. This resulted in the patient being even dizzier, with falls and breaks – a significant risk to patient safety, malpractice, and reduced trust in the system.

Another risk of such error occurred due to the manufacturer's misconfiguration of the e-prescribing system. There was an exchange of information between a system and a system component. One system was a standalone system with the software used for healthcare documentation (EMR), and the other was a common platform used for ‘drug handling’ (pharmaceutical module) that took place in real-time using multiple integration platforms. When an end-user opened the window in the EMR, it could remain open even though the user had changed patient in the pharmaceutical module, i.e., there was information from two different patients on the screen. It was possible to make a so-called exit to the system containing patient information via the pharmaceutical module (Microsoft Internet Explorer). The root cause of the error was that the EMR was not configured according to the instructions in the pharmaceutical module. This led to the risk of confusion between two different patients. Incorrect patient information could be documented in the other patient's medical record, which could further lead to inaccurate prescriptions being issued.

Another problem arose due to the lack of system configuration, which stated that doctors and pharmacists did not have the same view of the list of medicines. Medicines that were placed temporarily ‘on hold’ could not be seen. The pharmaceutical module showed an unauthorised distribution of drug administration, even though they were distributed according to prescription. Even though these problems were initially associated with drug handling errors, the analysis indicated that the pharmaceutical module of the EMR contributed to these errors. This error led to subsequent erroneous decisions by the doctor, entailing patient safety risks for multiple patients due to system issues. One patient death was reported; however, the death did not appear to be directly caused by inadequate drug handling or system configuration issues.

### Interface with other software systems or components

Continued lack of electronic coordination between the pharmaceutical module of the EMR's list of medicines and an electronic prescription tool for multi-dose drug dispensing was reported. The reports indicated several physicians’ complaints about the lack of electronic communication support, particularly after a software update, due to the shortcomings in the regional health authority's IT organisation. Similar software interface issues were reported between the EMR and the patient administration system involving the Care Portal, Care Documentation, and Care Administration system modules. The lack of system integration among these systems further complicated the pharmaceutical module to cause missing patient information regarding patient hypersensitivity to drugs and food. Among patient inconvenience and harm, these software problems resulted in delayed treatments or diagnoses, over-treatment, and under-treatment. The staff-related outcomes included extensive manual work and frustration among healthcare professionals. Overall, these issues led to reduced trust in the systems at the organisational level.

Software interface issues also involved the prescriptions’ validity period, which impacts the EMR and the pharmaceutical module. If the time between the last withdrawal and the prescription's last validity period was less than 3 months, the prescription was shown as expired in the list of prescriptions. Only active prescriptions, instead of ongoing treatment, were displayed when a patient was prepared for admission to inpatient care. Thus, medicines with expired prescriptions could easily be missed and therefore not prescribed and administered during a care session. This affected well-established working methods among care providers, with a risk of serious drug interactions for the patients. For example, there was a particular time when the patient and healthcare professionals believed the treatment to be no longer relevant. It was also possible that the drug was not prescribed and administered during a care session in inpatient care since the drugs were also not shown during preparation for patient admission to the hospital.

### Software functionality

Several software functionality issues occurred during day-to-day Swedish clinical practice. These involved sick certificates being sent but not received by the social insurance office; the exit (software) function of the medication list did not work, and patient medication information disappeared. These problems hindered the information and documentation exchange among different care units. Of these, two cases indicated that message delivery stopped causing delays in care delivery. Some of these problems posed the risk of serious deterioration in patients’ health due to delayed delivery of the patient data. Staff or organisational outcomes involved additional manual work and extra documentation for healthcare staff.

Several other cases described that electronic prescriptions were missing or lost. For example, a number of prescriptions for inpatient care disappeared for three patients. In a couple of cases, these incidents were considered to be serious large-scale events since multiple prescriptions vanished due to a functional issue (incorrect generation of prescription ID). These errors resulted in staff or organisational outcomes affecting healthcare efficiency and patient-related outcomes. Staff or organisational outcomes were associated with a negative impact on care delivery for multiple patients, such as back-and-forth follow-up, phone calls, additional documentation, and the use of extra resources. Patient-related outcomes included delays in care delivery and a risk that patient health would deteriorate.

### Data storage and backup

A number of prescriptions were missing from the mini-backup that was intended for use in the event of unplanned malfunction to maintain the integrity and reliability of patient data. There was a risk that the patient would not receive prescribed medication in the event of ongoing hospital care due to the missing data in the mini-backup.

### Record migration

A problem related to the new version of the pharmaceutical module resulted in information being lost, disappeared, and incorrect information being displayed. The upgrade to the latest version entailed migrating historical data from the legacy systems, posing challenges in maintaining data integrity after the launching process. This challenge posed the risk of incorrect dosing of drugs on the prescription due to ‘missing information’ and ‘incorrect information’, leading to severe medical damage.

### Software not accessible

A software functionality issue that occurred earlier was overlooked by the management, which meant that the staff did not have access to the vaccination file. The inaccessible software that handled the valid vaccination registration did not report any patient harm. However, delayed information that resulted from software problems caused patient inconvenience as treatment was delayed or involuntarily interrupted.

### Network/server down or slow

A network shutdown of the e-prescribing system caused the medication lists to not function for a long time. This caused frustration among the staff and a delay in care delivery for a number of patients. It was also reported that staff manually entered information into the system during the downtime of the system and server. In this case, staff and the organisation needed to use more resources, such as time and additional devices, to ensure the continuous healthcare process during the failures. However, no patient harm was reported in this category.

### Contributing (human versus technical) factors

There were a few consistent signals of things going wrong, where human factors or violations played a significant part in contributing to those incidents related to e-prescribing. Human failures or unawareness comprised factors associated with both healthcare professionals and patients. Issues related to human factors fell into a finite number of categories: prescriptions not cancelled actively by the pharmacist or prescriber; drug handling errors; staff unaware of a specific function in the national e-prescribing system; failure to sign dispensed medicine; staff inattention; a lack of knowledge of the software programming (code) developing by the software developer; and patients unable to remember previously prescribed medicines. All of these manifestations of human failure are represented in the three-step workflow process – transmission, storage, dispense, and use of electronic prescriptions.

On the other hand, technical factors were implicated in the genesis of the following finite categories: system misconfiguration; software/system/server malfunction; software programming error in the previous correction; system update/upgrade; system design issues, and system integration issues. Most of these contributing factors also deliver a similar message and have a common association with the issues discussed above. This phenomenon is called the ‘recursive’ nature of things that went wrong. An incident can be grouped under one or more contributing factors, and one contributor can trigger another or similar type of issue in the same incident. Thus, the manifestation of the recursive nature of narrations of things going wrong may be labelled as either incident type or contributing factor based on its salience in the specific context.

### Consequences and actions that are taken to mitigate the risks

The software issues (e.g., system design or updates) were manifested in the genesis of various consequences, such as incidents affecting multiple patients’ care management, major inconvenience to patients (delays), patient harm (risks of serious deterioration of health), and extra manual work for the healthcare staff. Even though these issues were potentially compliant with system redesign or tailoring the system to fit the current workflow, they were put in the ‘too hard basket’. The administrative arrangements included placing the dysfunctional ‘legacy’ system from one region to another without fixing the previously occurred technical issue. Thus, the system was continuously dysfunctional.

In other cases, the initiatives to solve the problems were merely ignored by the administrative personnel. The task of correcting the situation was taken after a long period, but the damage had already been done. For example, there were several cases of the risks for incorrect dosage of drugs since the user did not pay attention to improper programme behaviour of the system that remained unsolved for a relatively long period. The reports indicated that the incident investigation body did not receive any notification regarding system failure for several months. Later, it was found (after investigation) that the incorrect coding of the system was due to the system developer's lack of knowledge. The administrative adjustments were put in place until the algorithm was corrected after several months.

Some of the problems that have been described arose from systems and technical solutions outside of the healthcare organisation where the problems were detected. Issues included discrepancies between medication lists originating from the national e-prescribing system, insufficient interoperability between EMRs and the national prescription repository, and legal limitations. Thus, these problems must be resolved at a higher (national) level. As the core issues could not be resolved within the healthcare organisations experiencing them, other temporary initiatives or procedures were used to mitigate the problems. The procedures included cover letters to patients, discussions with local pharmacies to avoid misunderstandings, and the development of local and regional strategies to compensate for the apparent patient safety risks with the above structural problems.

## Discussion

The salient feature of a resilient system ensures that similar events are not repeated and that the system can detect and prevent harm to patients, staff, and the organisation as a whole.^43,44^ Despite the frequent and recurrent adverse events, most of the issues identified in this study are amenable to reduction, and improvements can be made with rigorous system planning, design, development, implementation, and management. In particular, this study exemplifies the usefulness and suitability of an incident reporting system that would orient researchers and analysts to track the evolving causes of electronic prescription-related patient and organisational harm. The analyses would further help to understand the exact underlying mechanisms of such issues, and where or at which stage (of the clinical workflow) the preventive and corrective strategies need to be directed.

Software and hardware issues are interconnected in many ways in healthcare. There remain challenges with slow and overflowing EMR comprising an overwhelmingly massive volume of data which is continuing to expand, unlike any other industry. The overwhelming volume of data means that new hardware is required, and the software systems used have to be faster and regularly upgraded. However, these may not be considered to be the best solution due to the limitations in the healthcare budget.^45^ Even though no hardware problems were detected in our findings, we assume that there would have been some hardware issues associated with the software problems, but these were not reported. Due to insufficient knowledge of the HIT issues by those reporting the problems and inadequate accounts of the incident reports, it was not possible to determine the underlying reasons or mechanisms.

Launching a new system or updating to a new version of any system or application entails migrating historical data from the legacy systems. These often pose challenges in maintaining data integrity during and after the introduction and launch of the new system. Studies also suggested that system or software updates were associated with decision support errors and delayed care, such as updating a drug database, which is consistent with our findings. System updates were linked to several other software-related issues, such as system configuration and software functionality issues.^46^ If these challenges are left undetected even for a shorter period, it can cause severe consequences for multiple patients and widespread impact at a system level. Financial implications also accompany these incidents; for example, investigating such events is highly cost-effective.^47^

In most cases, it's not always possible to devise preventive and corrective strategies for most of these system issues locally due to inadequate descriptions of the incident reports. The reporters are seldom aware of various system components and how those systems function. There was evidence in the reports that much time and effort was spent on alternative arrangements being made, such as manually handling data and refreshing or rebooting the systems. Details of such actions taken, including manual entry and computer restarts, do not convey any strong message but are an attempt to ensure that patient treatment can proceed without delay when the servers are down. These workarounds are common in healthcare practice and are often considered unworthy of an incident report in the majority of cases. Moreover, the frontline operators find it difficult and time-consuming to seek ‘fixes’ from the vendors or the system suppliers.

Jabin^48^ argued in their thesis that the technical problems at the system level might be apparent at any stage in the clinical workflow resulting in patient harm that requires system-level solutions. This means that the systems must be designed to prevent specific errors. Potential local solutions should be governed so that ‘early detection and mitigation’ play a vital role. Considerable thought and ingenuity will be needed with support and validation by studies, including observational and ethnographic studies. This approach will ensure the prevention and mitigation of errors in the context of the various clinical workflow stages at which they occur.

### Discrepancies with the medication list

Some of the issues encountered in the incidents collected for this study had their roots in the systems that were deployed. The problems were faced either at the care level in EMRs or at pharmacies in dispensing systems integrated with the national prescription repository. Poor interaction among the prescriber, care providers, and pharmacist resulted in frustration and challenging working conditions.

The findings in this study showed that different medication lists existed in different systems due to the lack of communication support between the systems. This posed a significant risk to patient safety. Because regional healthcare in Sweden is decentralised, Sweden introduced national projects, including the national patient overview (In Swedish: Nationell patientöversikt, NPÖ) and the National Medication List This was done to integrate medical information from the different regional authorities and HIT systems to provide healthcare professionals with a complete overview of the patients. The national patient overview, in general, allows authorised care staff to access patients’ medical records from any care provider in Sweden, regardless of the EMR. It aimed to handle issues involving system interface, minimise the interruption in healthcare delivery, and prevent potential patient harm.^49^ The National Medication List replaced the national prescription repository (shown in Figure 1) in May 2021 (after data collection for this study) but is currently only available through a web-based service until being integrated into EMR and other systems. This national initiative aims at providing healthcare professionals, pharmacies, and patients with the same information about an individual patient's prescribed and collected medicines.^49^ The expectations for the National Medication List are high, and it has the potential to improve many of the described problems and decrease discrepancies in medication lists. However, the National Medication List will only include medications with prescriptions to be collected from local pharmacies and will not include medications that are administered within healthcare, which will still only be visible in the individual EMRs. It is important to keep track of incidents related to medication management and the e-prescribing system during the ongoing implementation of the National Medication List.

The findings of our study indicated general practitioners’ reduced trust in the system due to an overwhelming lack of coordination among the HIT systems and electronic prescriptions. These issues further introduced clinical errors compromising patient safety and medication outcome, which had severe consequences. Ensuring coherence in the design and implementation of the EMR and e-prescribing systems, maintaining the channel of data integrity, and communication coordination between these systems may reconcile the clinical practice and improve patient safety.

Furthermore, a recent report on the population-wide system deployment of electronic prescriptions found failings in healthcare centres.^22^ The report indicated that 84% of the patients’ medication lists used by the general practitioners were not updated in the follow-up of the patients in a review of EMR at ten primary healthcare centres.^22^ Another study reflected on similar findings and found poor interoperability to be a considerable challenge for the integration of HIT systems into daily clinical practice.^50^ The participants in the study reported the discrepancy in electronic prescription and the challenges of HIT system integration, supplemented by the healthcare staff's inability to send data to the national patient overview.^50^ These findings are in synchrony with the results in this study; however, the numbers^22^ need to be treated with caution since they are not synchronised with the interpretation of our findings; instead, the nature of the challenges arising remains the same.

## Recommendations

We recommend the following implications for clinical practice based on the results obtained from this study and the considerations emerging from various literature and public reports. We believe these recommendations will help healthcare organisations, e-prescribing system developers, HIT experts, government agencies, researchers, analysts, policymakers, relevant stakeholders, and funders to improve healthcare quality and patient safety. These recommendations are categorised following the recent publication by Sittig et al.,^51^ with various stages of HIT lifecycle where they appear: (a) design and development, (b) implementation and use, and (c) monitoring, evaluation, and optimisation.^51^

These recommendations should be put in place to mitigate the risks associated with the design and developmental challenges.

- **Design standard user-interface features and functions-** The evidence in this study suggests that poor user-interface design led to errors between EMR systems and the components/modules of the e-prescribing system. For example, most medication lists in the pharmaceutical module of the EMR may have different views of presenting patients’ prescriptions. Such a deficiency should be handled by using better-standardised methods of data entry to ensure that data entry is correct by the end-user for a specific patient.^52^ The HIT industries should come forward to design and develop well-established standards for safety-critical software. These standards should be developed based on desired and appropriate working procedures, and they should be maintained by national and international standardisation bodies and endorsed by government authorities.
- **Ensure software quality in an interfaced, networked healthcare environment-** Lack of communication and appropriate configuration between systems have been the most important problems identified in this study. The issues of data integrity and the transfer of historical records to a new system have also been technically challenging, which were left undetected for a long period.^35,36^ Therefore, harmonising HIT systems is critically important, particularly when a component of the system, a version of the system, or the entire system is obtained from multiple vendors. For example, the configuration of the EMR should be in synchrony with the e-prescribing system for continuous management of the clinical workload. These systems should be interoperable and communicable, ensuring access to clinical studies or patient records to and from each other.^35,44^ Moreover, the complex nature of the healthcare system always brings about new HIT functionalities along with the modules/components of the system and the standalone applications. These system modules and applications must be interfaced appropriately with the existing system, and the whole process of interfacing must be error-free.^52^
- **Develop and design the systems that fit the clinical workflow-** Introducing a new HIT system has always been challenging from administrative and logistical aspects, particularly in a complex sociotechnical healthcare organisation.^35,53^ Since introducing a new system will be costly and disruptive, it is highly recommended that the end-users, including healthcare professionals (within and outside healthcare facilities), have some degree of ownership and management. Such system implementation should be user-friendly and be suited to the clinical workflow by engaging multiple actors, such as vendors, administrators, external consultants, corporations, and even politicians (if needed).^35,54^ Issues pertaining to healthcare quality should also be addressed in the process of building any software and deploying a new system, ensuring the prospective sound governance of the HIT systems.^54^
- **Design and develop proactive models and tools for evaluating risks-** A thorough understanding of things that go wrong and how they go wrong should be synchronised with the features and functions of the complex HIT system.^35,55^ For example, a patient receiving the wrong medication due to the wrong selection of a correct item or the correct selection of the wrong item should be identified to detect risks to patient safety. This can be achieved by retrospective incident analysis and the use of expert opinion to determine the severity and likelihood of potential risks with an event.^34^ Therefore, we need a proactive data-driven model to assess the seriousness and frequency of incidents to prevent or at least mitigate the chances of those events from occurring. This will be useful to identify the challenges automatically within the digital systems, such as EMR and its associated system component, including the e-prescribing system.^56^

These recommendations will reduce the challenges regarding implementation and use.

- **Plan carefully in HIT system transitions-** Careful consideration in deploying a new system or scheduling HIT system changes must be prioritised. Such planning will require close cooperation among the vendors of both the existing and old systems to ensure the appropriate integration of new HIT systems in the healthcare centre.^44^ For example, the National Medication List should be correctly integrated with the existing EMR by understanding the needs of the healthcare services and each system about how they function and how their functional capacity is incorporated with the healthcare centre. Synchronisation of various HIT systems and exposure indicators is essential, particularly when products or versions of the products emerge from multiple vendors. This harmony can be achieved through continuous consultation among the HIT vendors, HIT specialists, the end-users, and the researchers involved in maintaining and monitoring such systems.^35^ This will eventually reduce the risks associated with the system design issues and issues related to the software interface and system integration problems.
- **Set up a training process for the frontline operators-** Providing training and education to healthcare professionals,^57,58^ such as physicians, prescribers, and pharmacists, ideally in cooperation with HIT vendors, will minimise the risks associated with user error. The study by Saner and van der Velde^50^ also proposed that additional courses and training are needed for the users of HIT systems to align with the national project. A continuous training programme should be incorporated into HIT vendor contracts, and the professional development of the clinical staff should be introduced by ensuring funding for adequate paid time.^35^
- **Establish consumers’ health literacy programme-** Another way of mitigating errors in use is to ensure consumers’ basic health literacy and involve patients in the management of their own safety.^59^ For instance, a defence mechanism in the broader healthcare system can be enhanced by detecting the wrong prescription (either for another patient or incorrect drugs) with the help of providing basic health literacy to patients.^60^
- **Ensure adequate funding for local fixes and training processes-** No matter what strategies are undertaken, the risks associated with the system issues, including system error, upgrades, and functionality issues, always exist in complex healthcare. Many of the incidents in this study were repetitive, yet no steps were taken to prevent them as part of the proper maintenance of HIT systems at the local level. An aspect of having great worth is to ensure sufficient funding for incorporating software updates or system upgrades for local fixes.^35^

These solutions are recommended at the system-wide level to minimise the challenges related to monitoring, evaluation, and optimisation.

- **Develop a patient-staff HIT safety model-** Since the healthcare responsibility lies with both patients and care providers, there should be a model or method that would minimise the HIT risks to benefit both these groups. For example, patients should be able to report any mishap to their physicians in the event of medication order errors experienced at the pharmacy.^61^ On the other hand, physicians should be able to take responsibility for mitigating such errors with the help of activity tracking and personal/shared health records.^62^ This approach may be challenging as it requires a balanced cultural shift, but possible, with a new degree of transparency entailing accessibility of additional clinical data and progress notes.^63^
- **Ensure safety standards for the e-prescribing system at a national level-** The current e-prescribing systems in healthcare are not liable to any established clinical safety standard, which had the obvious potential to be under operational oversight of the software/hardware used in the care delivery. Reinforcing safety standards at the population level for the sound maintenance of the e-prescribing system is the only way to minimise the risks associated with repeatedly occurring system design and user interface issues in healthcare.^54,64^
- **Respond to problems at a population level-** The robust mechanism should also include identifying clusters of electronic prescription-related incidents that need prioritising to ensure that urgent preventive and corrective interventions are applied at a population level.^48^ The prioritisation of such incidents can be achieved by providing ‘cues’ in the reporting system, like those used in the HIT-CS, for the completeness of the report. Working on refining the current reporting systems and the system-wide approach with the ‘cues’ are ideal for effective reporting with fewer incidents that need due attention for immediate feedback.^65^
- **Ensure human surveillance-** The continuing refinement of professional development, training, and safeguards in the safe use of HIT systems should be accompanied by the existing endorsement of healthcare organisations’ safety practices. For example, verification of user proficiency in operating the systems should be introduced at the organisational level. The certification should establish regular renewal of the operators’ credentials, specifically in the event of any software updates or changes in the workflow.^32^

### Strengths and limitations of the study

The incidents studied in this article were voluntary or self-reports with inherent limitations, including bias with respect to reporting incidents that may emerge as interesting or unusual or that occur, repetitively.^53,66^ This acknowledges the reporters’ lack of knowledge of the HIT systems or willingness to provide detailed reports. However, the incidents were reported over a 5-year period, providing adequate and useful data for a profile of the issues encountered until the interventions are introduced as preventive and corrective strategies. Although no cause-and-effect relationships can be drawn firmly from this time period, the emergence of new problems is constantly on the agenda due to the complex nature of healthcare.^66^

Data collection was designed based on the convenience of the potential participants and the scope of the study due to the ongoing coronavirus disease 2019 pandemic worldwide. The participants could respond either by email (written) or telephone (semi-structured interview). In addition, participants were also asked to provide more retrospectively collected incidents from the local database (if possible). A follow-up communication was conducted to gain more up-to-date information about things that went wrong to extract additional information about the context, any regularly used recovery strategies, and how incidents might have been prevented or mitigated

There is no doubt that the response rate is low and that the sample size is small with regard to the e-prescription incidents. However, the collection of e-prescription-related incidents was rich in narrative texts and described significant risks to patient safety that required special attention. This is why the focus should have been made on minor incidents related to electronic prescriptions despite the limited sample size. Moreover, the small sample size also made it possible to ensure the validity of the search term in identifying the targeted incident reports. However, the results must be considered with caution because not all of the incidents were representative of the category for healthcare quality or patient safety events. It was impossible to determine the contributing factors or degree to which they were associated with the type of issues in a few cases.

Medication errors or issues related to the e-prescribing system are common in countries such as the USA,^67,68^ Australia,^69,70^ the UK,^71–73^ and European countries, such as Denmark,^26^ Norway^25^ and the Netherlands.^28^ Like many other countries, Sweden is no exception to being prone to these challenges; however, limited attempts have been made to overcome these issues since most reported events are repetitive, causing reduced trust in the healthcare system. For example, a systematic review of the HIT systems and their effects on the delivery of care by Kim et al.^74^ found around half of the included studies experienced problems related to medication errors or electronic prescriptions. This suggests that the results and lessons learned can be useful and applicable elsewhere for patient safety and healthcare quality improvement studies.

## Conclusions

Healthcare quality and safety of the HIT systems must be substantially improved and ensured. The source of information about things going wrong is one of the most reliable ways of identifying the HIT system challenges in routine clinical practice. Although scientific knowledge has constantly been improving, we believe that much more needs to be learned, and much remains to be explored to address these problems and strengthen HIT safety. Despite system improvement initiatives, such as the national patient overview and the National Medication List, many problems still remain. There is a clear need for further national and regional initiatives to improve medication management and the e-prescribing system. In addition, it is vital to monitor any issues and incidents arising from these large-scale implementations in the critical medication management process to identify new and remaining risks. This study focused on the issues encountered by the e-prescribing system, factors contributing to the problems, consequences faced by the patient, staff, and organisation, and actions taken to mitigate those risks through multiple qualitative approaches. Therefore, healthcare organisations using the e-prescribing system should adopt the recommendations devised in this study to overcome the challenges of improving healthcare quality and safety.
